# LNCAROD is stabilized by m6A methylation and promotes cancer progression via forming a ternary complex with HSPA1A and YBX1 in head and neck squamous cell carcinoma

**DOI:** 10.1002/1878-0261.12676

**Published:** 2020-04-13

**Authors:** Yuanyuan Ban, Pingqing Tan, Jing Cai, Junjun Li, Meng Hu, Ying Zhou, Yan Mei, Yixin Tan, Xiaoling Li, Zhaoyang Zeng, Wei Xiong, Guiyuan Li, Xiayu Li, Mei Yi, Bo Xiang

**Affiliations:** ^1^ NHC Key Laboratory of Carcinogenesis Hunan Cancer Hospital and The Affiliated Cancer Hospital of Xiangya School of Medicine Central South University Changsha China; ^2^ Hunan Key Laboratory of Nonresolving Inflammation and Cancer The Third Xiangya Hospital Central South University Changsha China; ^3^ The Key Laboratory of Carcinogenesis and Cancer Invasion of the Chinese Ministry of Education Cancer Research Institute and School of Basic Medical Sciences Central South University Changsha China; ^4^ Department of Dermatology The Second Xiangya Hospital The Central South University Changsha China; ^5^ Department of Dermatology Xiangya Hospital Central South University Changsha China

**Keywords:** epitranscriptome, molecular chaperone, protein–RNA interactions, RNA modification, ubiquitin–proteasome pathway

## Abstract

Head and neck squamous cell carcinoma (HNSCC) constitute approximately 4% of all cancers worldwide. In this study, we analyzed the expression profile of the long noncoding RNA (lncRNA) of 502 HNSCC patients from The Cancer Genome Atlas database. Among the differentially expressed lncRNAs between HNSCC and normal samples, LNCAROD is overexpressed in HNSCC and associated with advanced T stage and shortened overall survival. The *N*6‐methyladenosine (m6A) modification mediated by METTL3 and METTL14 enhanced the stability of LNCAROD in HNSCC cells. Depletion of LNCAROD attenuated cell proliferation, mobility *in vitro*, and tumorigenicity *in vivo*, whereas overexpression of LNCAROD exerted opposite effects. LNCAROD is mainly distributed in nucleus and binds with YBX1 and HSPA1A proteins. Silencing either YBX1 or HSPA1A did not affect the level of LNCAROD. However, loss of LNCAROD led to shortened half‐life of YBX1 protein. Mechanistically, LNCAROD protected YBX1 from proteasomal degradation by facilitating YBX1‐HSPA1A protein–protein interaction. Depletion of HSPA1A in LNCAROD‐overexpressing cells resulted in accelerated proteasomal degradation of YBX1 protein. Moreover, re‐expression of Flag‐YBX1 in LNCAROD‐silenced cells rescued malignant behavior of HNSCC cells. Our study indicates that LNCAROD is an oncogenic lncRNA and dysregulation of m6A modification might account for aberrant expression of LNCAROD in HNSCC. LNCAROD acts as a scaffold for the interaction between YBX1 and HSPA1A, preventing proteasomal degradation of YBX1 in HNSCC cells.

## Graphical abstract

Dysregulation of long noncoding RNAs (lncRNAs) is implicated in cancer development and progression. In this study, Ban *et al*. demonstrated that an oncogenic lncRNA, LNCAROD, is stabilized by m6A methylation and overexpressed in head and neck squamous cell carcinoma (HNSCC). LNCAROD forms a ternary complex with HSPA1A and YBX1, preventing proteasomal degradation of YBX1. This study provides a new paradigm of the mechanisms of lncRNAs in HNSCC development.

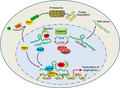

**Abbreviations**
ASO: antisense oligonucleotide
CCK‐8: cell counting kit‐8
HNSCC: head and neck squamous cell carcinoma
HSPA1A: heat‐shock 70‐kDa protein 1A
LNCAROD: lncRNA‐activating regulator of DKK1
LncRNA: long noncoding RNA
m6A: *N*6‐methyladenosine
METTL14: methyltransferase‐like 14
METTL3: methyltransferase‐like 3
OS: overall survival
RIP: RNA immunoprecipitation
TSCC: tongue squamous cell carcinoma
YBX1: Y box binding protein 1

## 1 Introduction

Head and neck cancer is an aggressive life‐threatening malignancy, with > 830 000 estimated new cases and > 430 000 estimated death per year worldwide (Bray *et al.*, 2018). Head and neck squamous cell carcinoma (HNSCC) accounts for the majority of head and neck cancer. Several risk factors link to the development of HNSCC, including consumption of tobacco and alcohol (Blot *et al.*, 1988; Maier *et al.*, 1992) and infection of human papillomavirus (Chaturvedi *et al.*, 2011; Cramer *et al.*, 2019). During the past decades, intensive studies have been performed to explore the molecular mechanisms underlying the development and progression of HNSCC, most studies focus on protein‐coding genes. Long noncoding RNAs (lncRNAs) are defined category of RNAs longer than 200 nt without protein‐coding or with limited protein‐coding ability (Raj and Rinn, 2019). LncRNAs have been implicated in multiple physiological processes and also contribute to development of various diseases, including cancers (Yao *et al.*, 2019). Many lncRNAs distribute in the nucleus and associate with chromatin and are implicated in chromatin remodeling and transcription regulation (Tang *et al.*, 2017; Yamamoto and Saitoh, 2019). A considerable amount of cytoplasmic lncRNAs have been identified and are linked to various biological processes, including sponging of miRNAs, translation of mRNAs, protein stability and modulation of metabolisms and cell signaling transduction (Fan *et al.*, 2017; Noh *et al.*, 2018; Tang *et al.*, 2018).

Apart from transcriptional control, various epitranscriptomic modifications expand the variety of transcriptomes. *N*6‐methyladenosine (m6A)methylation is the most abundant and most well‐studied one. Abnormal levels of m6A modification are implicated in human cancers, including acute myeloid leukemia (Barbieri *et al.*, 2017; Vu *et al.*, 2017; Weng *et al.*, 2018) and glioblastoma (Visvanathan *et al.*, 2019, 2018, 2019, 2018). The m6A modification affects various aspects of RNA metabolism, including structure, maturation, stability, splicing, export, translation, and decay (Chen *et al.*, 2019; Lan *et al.*, 2019). To date, most of the studies focus on effects of m6A modification on mRNAs. The complex molecular epitranscriptomic events associated with noncoding genes and their contribution to the initiation and progression of HNSCC still remain elusive.

Here, we showed a lncRNA, LNCAROD, is overexpressed in HNSCC and associated with advanced T stage and poor prognosis. LNCAROD is stabilized with m6A methylation in HNSCC cells. Moreover, we found LNCAROD serves as a scaffold to facilitate YBX1‐HSPA1A protein–protein interaction, there for preventing proteasomal degradation of YBX1. LNCAROD promotes HNSCC cell proliferation and mobility through stabilizing YBX1 protein.

## 2 Materials and methods

### 2.1 LncRNA expression profile and clinical data

The RNA expression profiles and clinical data of HNSCC were obtained from the TCGA database (https://cancergenome.nih.gov/). A cohort including 502 HNSCC samples and 44 normal samples were used in this study. Human lncRNA genes stable ID was obtained from Ensembl database (http://asia.ensembl.org/index.html). The lncRNA expression profiles of HNSCC were extracted from RNA‐sequencing data of HNSCC. Differentially expressed lncRNAs between HNSCC and normal tissues were determined by r software (http://www.r-project.org/). The differentially expressed lncRNAs are listed in Appendix S1. The association of lncRNA expression to patients' overall survival (OS) was analyzed by Kaplan–Meier plotter (http://kmplot.com/analysis/index.php?p=service%26cancer=pancancer_rnaseq) based on TCGA data.

### 2.2 Clinical specimens

The use of clinical samples was approved by the Institute Research Ethics Committee of the Central South University, and each patient signed a consent form to participate in the study. Nineteen pairs of fresh cancer tissues and normal counterpart tissues from diagnosed HNSCC patients, including oral squamous cell carcinoma (OSCC), tongue squamous cell carcinoma (TSCC), and hypopharyngeal SCC, were collected from the Cancer Hospital of Hunan Province (Changsha, China) by surgery. None of the patients received any antitumor therapy before surgery. This study has been conducted in accordance with the Declaration of Helsinki.

### 2.3 Cell lines, cell culture, and transfection

HK1, FaDu, Tca8113, CAL‐27, NP69, and C666‐1 cell lines were used in this study. HK1 is a well‐differentiated SCC cell line of nasopharynx (Huang *et al.*, 1980), whereas NP69 is an immortalized nasopharyngeal epithelial cell line (Tsao *et al.*, 2002), and C666‐1 is an undifferentiated nasopharyngeal carcinoma cell line (Cheung *et al.*, 1999). FaDu is a human hypopharyngeal SCC cell line (Hegde *et al.*, 2019), and Tca8113, CAL‐27 cells are human TSCC cell lines. All these cell lines were routinely grown in RPMI 1640 medium (Gibco, Waltham, MA, USA), supplemented with 10% fetal bovine serum and antibiotics (100 units·mL^−1^ penicillin and 100 mg·mL^−1^ streptomycin). Cells were incubated at 37 °C in a humidified atmosphere of 5% CO_2_ in air. Transfection of siRNAs or antisense oligonucleotides (ASOs) was performed by using Lipofectamine RNAiMax reagent according to the manufacturer's protocol. Sequences of siRNAs and ASOs used in this study are listed in Table S1.

### 2.4 Cell counting kit‐8 assays

Tumor cells were seeded into 96‐well plates at a density of 2000 cells per 0.2 mL and allowed to grow for indicated time. Cell growth was measured by cell counting kit‐8 (CCK‐8) assays according to previous demonstration (Li *et al.*, 2019).

### 2.5 Colony formation assay

Tumor cell survival was measured by colony formation assays. Briefly, single‐cell suspensions were seeded into 6‐well plates at a density of 2000 cells per well and allowed to grow for 10–14 days. Visible cell colonies were fixed with methanol for 15 min and visualized by 0.1% crystal violet. Colony number was manually counted. Each assay was performed in triplicate.

### 2.6 Cell migration and invasion assays

Tumor cell mobility and invasiveness were determined by using 8‐μm‐pore Transwell inserts (Corning‐Costar, Cambridge, MA, USA) precoated without or with 15 μL Matrigel (BD Biosciences, Bedford, MA, USA) as described previously (Li *et al.*, 2019). Briefly, tumor cell suspensions in serum‐free medium at a density of 5 × 10^4^ to 10 × 10^4^ cells per 0.2 mL were seeded onto Transwell inserts. The inserts were placed in a lower chamber containing 600 μL of complete culture medium. Tumor cells were allowed to move across the Transwell insert membranes for 6–24 h at 37 °C. Migrated or invaded tumor cells were fixed and visualized by 0.1% crystal violet. The numbers of migrated or invaded cells were counted from five random fields.

### 2.7 RNA extraction, reverse transcription, and quantitative PCR

Total RNAs from tissue samples and cells were extracted by using TRIzol reagent (IInvitrogen, Carlsbad, CA, USA). After digestion with DNase (Takara, Beijing, China) to remove trace amount of genomic DNA, complementary DNA was synthesized with 1 μg of RNA by using reverse transcription kit (Thermo Fisher Scientific, Beijing, China) for lncRNAs and mRNAs. For lncRNAs, random primers were used for reverse transcription. Quantitative PCR was performed with 2× SYBR Green qPCR Master Mix reagents (Bimake, Shanghai, China) according to the protocol from the manufacturer on Bio‐Rad CFX–Connect™ Real‐Time PCR Detection System (Bio‐Rad, Richmond, CA, USA). The relative expression levels were counted according to $2^{-\Delta \Delta C_{T}}$ methods (Livak and Schmittgen, 2001). Sequences of specific primers for lncRNAs and mRNAs are listed in Table S2.

### 2.8 Biotin RNA–protein pull‐down assay and mass spectrometry analysis

The full‐length sense and antisense of LNCAROD RNA or its fragments (1–250, 1–500, 1–750, and 751–972 nt) were *in vitro* transcribed by using MEGAscript™ T7 Transcription Kit (Thermo Fisher Scientific, Waltham, MA, USA). Pierce™ RNA 3′ End Desthiobiotinylation Kit (Thermo Fisher Scientific) was explored to label the prepared transcripts with biotin *in vitro*. Biotinylated transcripts were mixed with whole lysates from HK1 cells, and RNA–protein complex was enriched with streptavidin beads (Thermo Fisher Scientific; Lot R12316318) according to the manufacturer's protocol. Mass spectrometry was performed by PTM Biolabs (Hangzhou, China).

### 2.9 Western blot

Total cellular proteins were extracted with lysis buffer supplemented with Protease Inhibitor Cocktail (Bimake). Protein samples were separated in 10–12% SDS/PAGE and transferred to PVDF membranes (Merck Millipore, Billerica, MA, USA). After blocking in 5% defatted milk, the membranes were incubated with primary antibodies overnight at 4 °C. Following incubation with secondary antibodies, signals were detected using the ECL detection system (Thermo Fisher Scientific). The following antibodies used in western blotting assays, HSPA1A (ABclonal Technology, Woburn, MA, USA; A3921), (Abcam, Cambridge, MA, USA; ab76149), Flag (Proteintech, Chicago, IL, USA; 2B3C4), H3 (Cell Signaling Technology, Beverly, MA, USA; D1H2), GAPDH (ABclonal Technology; AC033), normal rabbit IgG (Chemicon, Temecula, CA, USA; 2519253), and normal mouse IgG (Millipore; 12‐371).

### 2.10 RNA immunoprecipitation

RNA immunoprecipitation (RIP) assays were performed as described previously. Briefly, HK1 cells were lysed by using GLB buffer [Tris/HCl 10 mm (pH 7.5), NaCl 10 mm, EDTA 10 mm, Triton X 0.5%, DTT 1 mm, PMSF 10 mm, protease inhibitor cocktail]. Cell lysates were precleaned with recombinant protein A/G agarose (GenScript, Nanjing, China) to minimize nonspecific binding for 30 min at 4 °C. One to ten percent of the samples were used as input. Equal amount of cell lysates were incubated with YBX1 antibody or HSP1A1 antibody or normal rabbit IgG overnight at 4 °C. The RNA–protein/antibody complexes were captured by incubation with recombinant protein A/G agarose (GenScript, Nanjing, Jiangshu, China). For m6A RNA methylation assays, cellular RNAs were enriched by anti‐*N*6‐methyladenosine (m6A) antibody (Abcam; ab151230). RNA was extracted from the precipitated complex and transcribed into cDNA. RT‐PCR assays were performed to detect binding of RNA to proteins or antibody.

### 2.11 Pulse‐chase assays

For measurement of the half‐life of LNCAROD, actinomycin D (Sigma, St. Louis, MO, USA) was supplemented to the culture medium of HK1 cell. Total cellular RNAs were extracted by using TRIzol reagent at indicated time points. RNA level was measured by RT‐PCR as described above. For measurement of the half‐life of YBX1 protein, cycloheximide (CHX; Sigma) was added to the culture medium of HK1 cell. Cellular proteins were prepared by lysis buffer supplemented with Protease Inhibitor Cocktail (Bimake) at indicated time points. Protein level was then determined by western blot.

### 2.12 Statistical analysis

The association of LNCAROD expression with clinicopathological characteristics of HNSCC patients was analyzed using the Pearson chi‐square method. Quantitative variables differences between groups were analyzed by using Student's *t*‐test. A two‐way ANOVA was used to analyze the cell viability assay or growth curve. The spss 13.0 software package (SPSS, Chicago, IL, USA) was employed for statistical analysis. A value of *P* < 0.05 was recognized as statistically significant.

## 3 Results

### 3.1 LNCAROD is overexpressed in HNSCC and associated with advanced T stage and poor prognosis

**Fig. 1:**
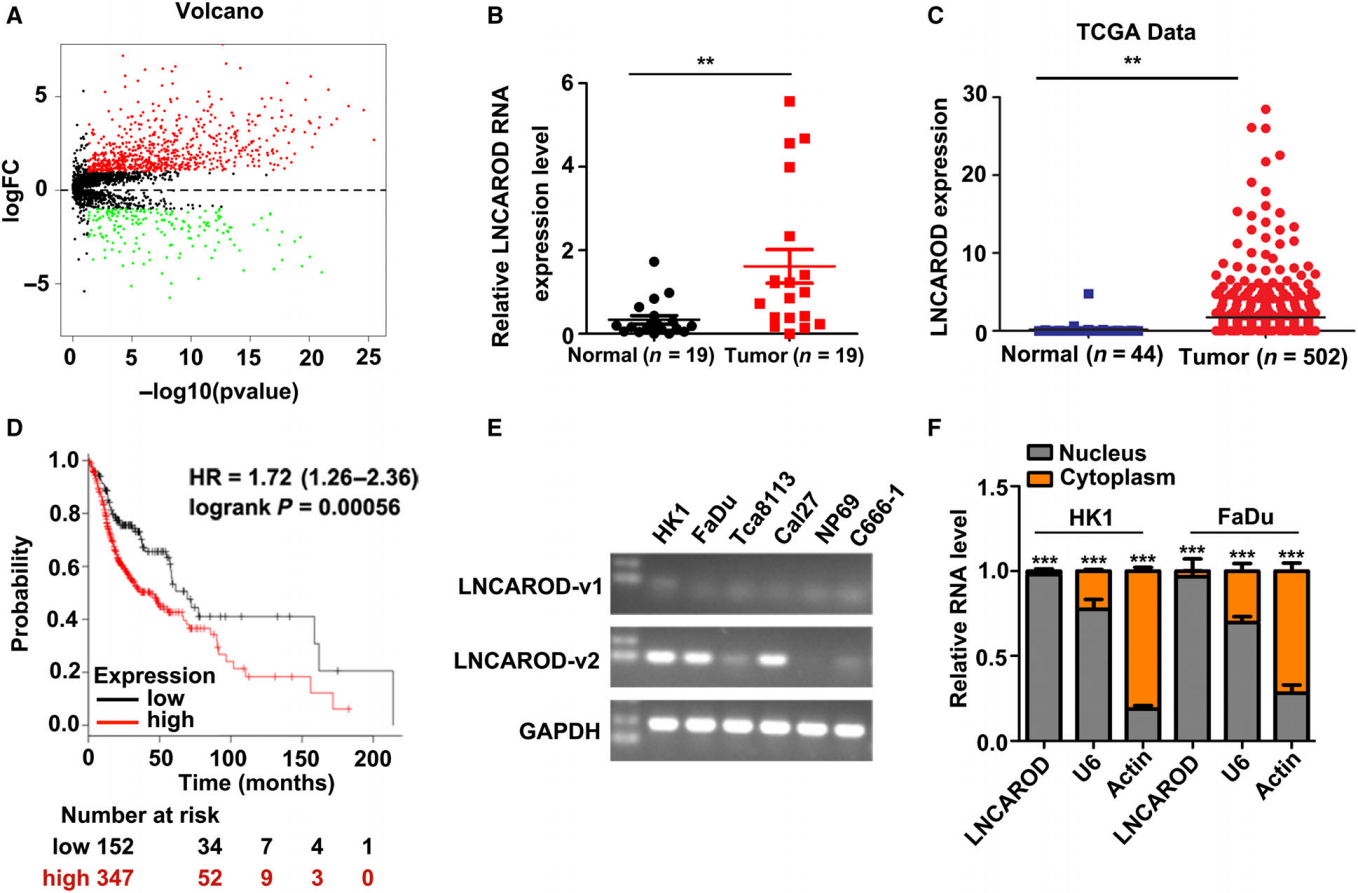
LNCAROD is overexpressed in HNSCC and predicts unfavorable clinical outcome. (A) Volcano plot described that the differentially expressed lncRNAs in HNSCC. (B) Measurement of LNCAROD expression level by RT‐qPCR assay in fresh HNSCC and normal tissues. (C) LNCAROD is upregulated in HNSCC samples according to TCGA dataset. (D) High level of LNCAROD predicts shortened OS in HNSCC patients. (E) Expression levels of LNCAROD variants in HNSCC cell lines, and normal cells were determined by RT‐PCR assay. (F) RT‐qPCR assay indicated that LNCAROD mainly distributes in nucleus fraction (*n* = 3 per group). All data are mean ± SD. Data were analyzed by using Student's *t*‐test. **P < 0.01, ***P < 0.001.

LncRNAs list was downloaded from Ensembl database (http://asia.ensembl.org/index.html). RNA‐seq data of HNSCC and normal samples were screened by using r software, with a criteria of fold change > 2, *P* < 0.05 (Fig. S1). The differentially expressed lncRNAs were displayed by volcano plots (Fig. 1A). Among those upregulated lncRNAs, lncRNA‐activating regulator of DKK1 (LNCAROD) (also named aslnc‐MBL2‐4 or LINC01468) was found to be overexpressed in HNSCC samples, which is in consistent with a previous report (Gao *et al.*, 2014). LNCAROD is also highly expressed in bladder urothelial carcinoma and predicts unfavorable prognosis (Gao *et al.*, 2019), suggesting an oncogenic role in cancer development. Thus, it was chosen for further validation. RT‐PCR assay showed that the level of LNCAROD significantly increased in HNSCC samples as compared to normal tissues (Fig. 1B). Then, we analyzed its expression in TCGA dataset. The results consistently showed that LNCAROD is increased in HNSCC samples (Fig. 1C). Although expression of LNCAROD is not associated with age and gender of HNSCC patients, high level of LNCAROD is associated with histological grade and positively associated with advanced T stage (Table 1). Moreover, we found that high expression of LNCAROD predicts poor overall prognosis of HNSCC (Fig. 1D). RT‐PCR also showed that LNCAROD variant 2 is highly expressed in several SCC cells from head and neck, including HK1, FaDu, and CAL‐27 cells, but weak in Tca8113 and C666‐1 cells, and absent in NP69 cell. RT‐qPCR assay indicated that LNCAROD is predominantly distributed in nucleus. Thus, our finding suggests LNCAROD might contribute to the development of HNSCC.

**Table 1:** Relationship between LNCAROD expression levels and clinicopathological parameters of HNSCC.

Variable	No. of patient	LNCAROD expression (%)		*P* value
		High expression	Low expression	
Age (year)				
> 60	254	181 (71.3)	73 (28.7)	
≤ 60	244	166 (68)	78 (32)	0.246
Sex				
Female	133	97 (72.9)	36 (27.1)	
Male	366	250 (68.3)	116 (31.7)	0.189
Histological grade (WHO)				
G1–2	360	268 (74.4)	92 (25.6)	
G3–4	120	74 (61.7)	46 (38.3)	0.006
Clinical stage				
I + II + III	195	137 (70.3)	58 (29.7)	
IV	304	210 (69.1)	94 (30.9)	0.430
pT status				
T1–2	177	112 (63.3)	65 (36.7)	
T3–4	312	227 (72.8)	85 (27.2)	0.019
Lymph node metastasis				
No metastasis	241	175 (72.6)	66 (27.4)	
Metastasis	240	157 (65.4)	83 (34.6)	0.054
Distant metastasis				
No metastasis	475	332 (69.9)	143 (30.1)	
Metastasis	5	3 (60)	2 (40)	0.477

### 3.2 The m6A modification stabilizes LNCAROD in HNSCC

**Fig. 2:**
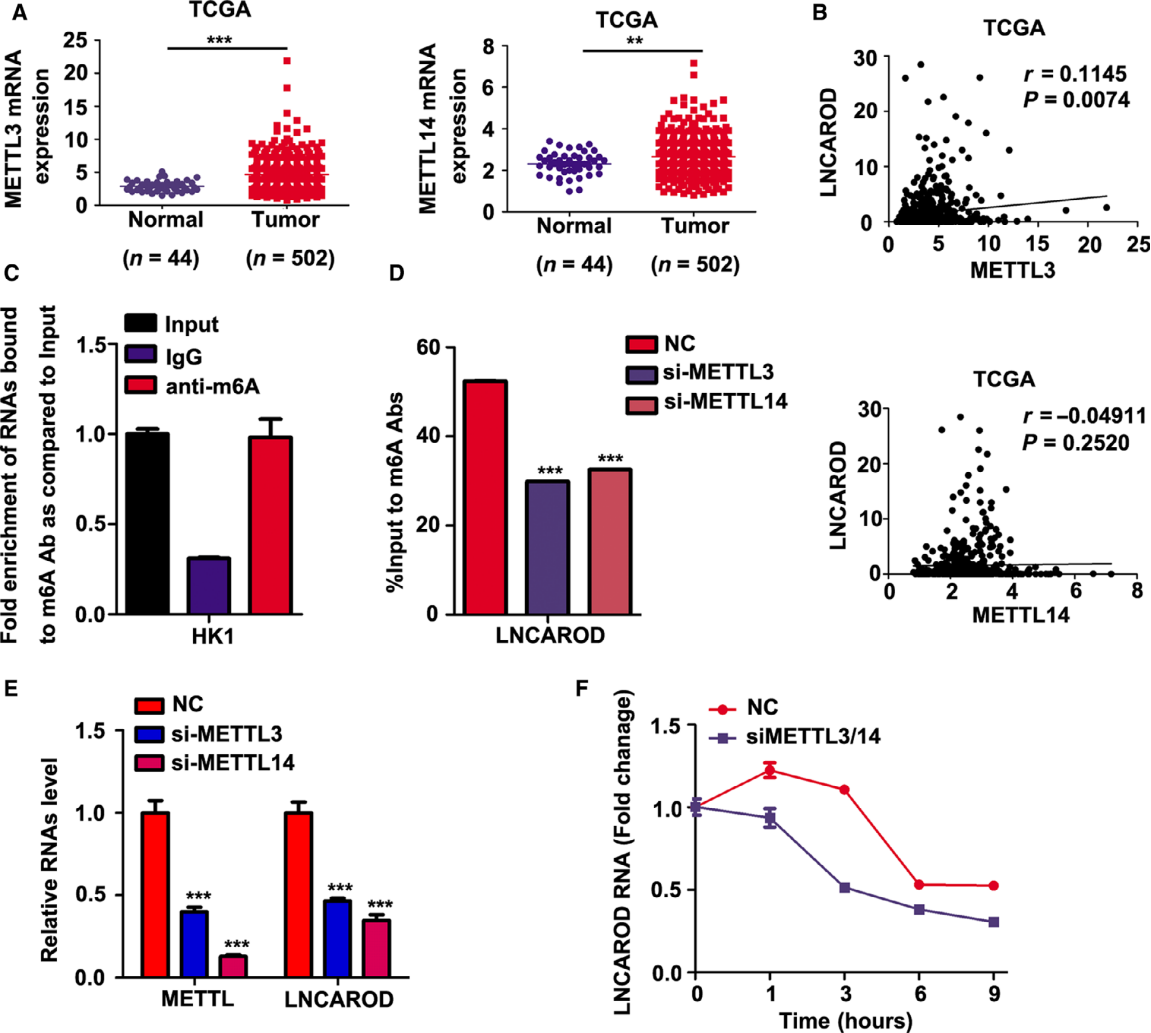
m6A modification catalyzed by METTL3 and METTL14 increases LNCAROD stability in SCC cells. (A) The mRNAs levels of METTL3 and METTL14 are elevated in HNSCC according to TCGA dataset. (B) Correlation analysis of LNCAROD with METTL3 or METTL14 in HNSCC samples. (C) RIP‐PCR assays performed by using m6A antibody suggested that LNCAROD is m6A modified in HK1 cell (*n* = 3 per group). (D) Depletion either METTL3 or METTL14 led to reduction of m6A modification level of LNCAROD (*n* = 3 per group). (E) Silencing either METTL3 or METTL14 resulted in downregulation of LNCAROD level in HK1 cells (*n* = 3 per group). (F) Pulse‐chase assay suggested that silencing METTL3 and METTL14 shortened the half‐life of LNCAROD. All data are mean ± SD. Data were analyzed by using Student's *t*‐test. **P < 0.01, ***P < 0.001.

The m6A modification of noncoding RNAs is emerging as a fundamental role in their regulation and functions (Coker *et al.*, 2019). We found that RNA levels of the RNA m6A methyltransferase METTL3 and METTL14 were upregulated in HNSCC samples according to TCGA dataset (Fig. 2A). Interestedly, high expression of METLL3 is positively correlated with the level of LNCAROD in HNSCC (Fig. 2B), prompting us to consider that m6A methylation contributes to dysregulation of LNCAROD in HNSCC. We performed pull‐down assay by using specific m6A antibody. We found that LNCAROD is significantly enriched in fraction immunoprecipitated by m6A antibody (Fig. 2C), indicating that endogenous LNCAROD is largely m6A modified in HK1 cell. When METTL3 and METTL14 were simultaneously depleted in HK1 cells, the level of LNCAROD dramatically decreased, which is accompanied with reduction of m6A methylation level of LNCAROD (Fig. 2D). Silencing either METTL3 or METTL14 leads to moderate decrease of LNCAROD level (Fig. 2E). Moreover, pulse‐chase assay showed that loss of METTL3 and METTL14 significantly shortened the half‐life of LNCAROD (Fig. 2F), suggesting that m6A methylation enhances the stability of LNCAROD.

### 3.3 LNCAROD promotes HNSCC cell proliferation and mobility *in vitro*

**Fig. 3:**
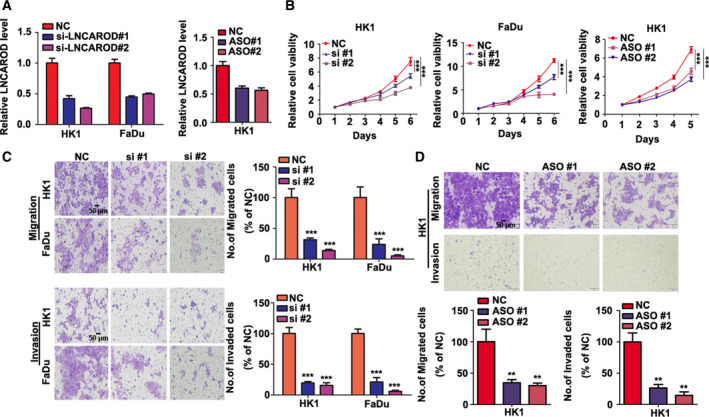
Transient silencing LNCAROD attenuates growth, migration, and invasion of HNSCC cells *in vitro*. (A) RT‐qPCR assays indicated that specific siRNAs or ASOs suppressed LNCAROD RNA levels in HK1 and FaDu cells (*n* = 3 per group, data were analyzed using Student's *t*‐test). (B) Growth of HK1 and FaDu cells transfected with siRNA or ASO were measured by CCK‐8 assays (*n* = 5 per group. Data were analyzed using two‐way ANOVA). (C, D) Cell migration and invasion assays were performed by using transwell inserts assays (*n* = 3 per group. Data were analyzed using Student's *t*‐test). All data are mean ± SD. **P < 0.01, ***P < 0.001.

**Fig. 4:**
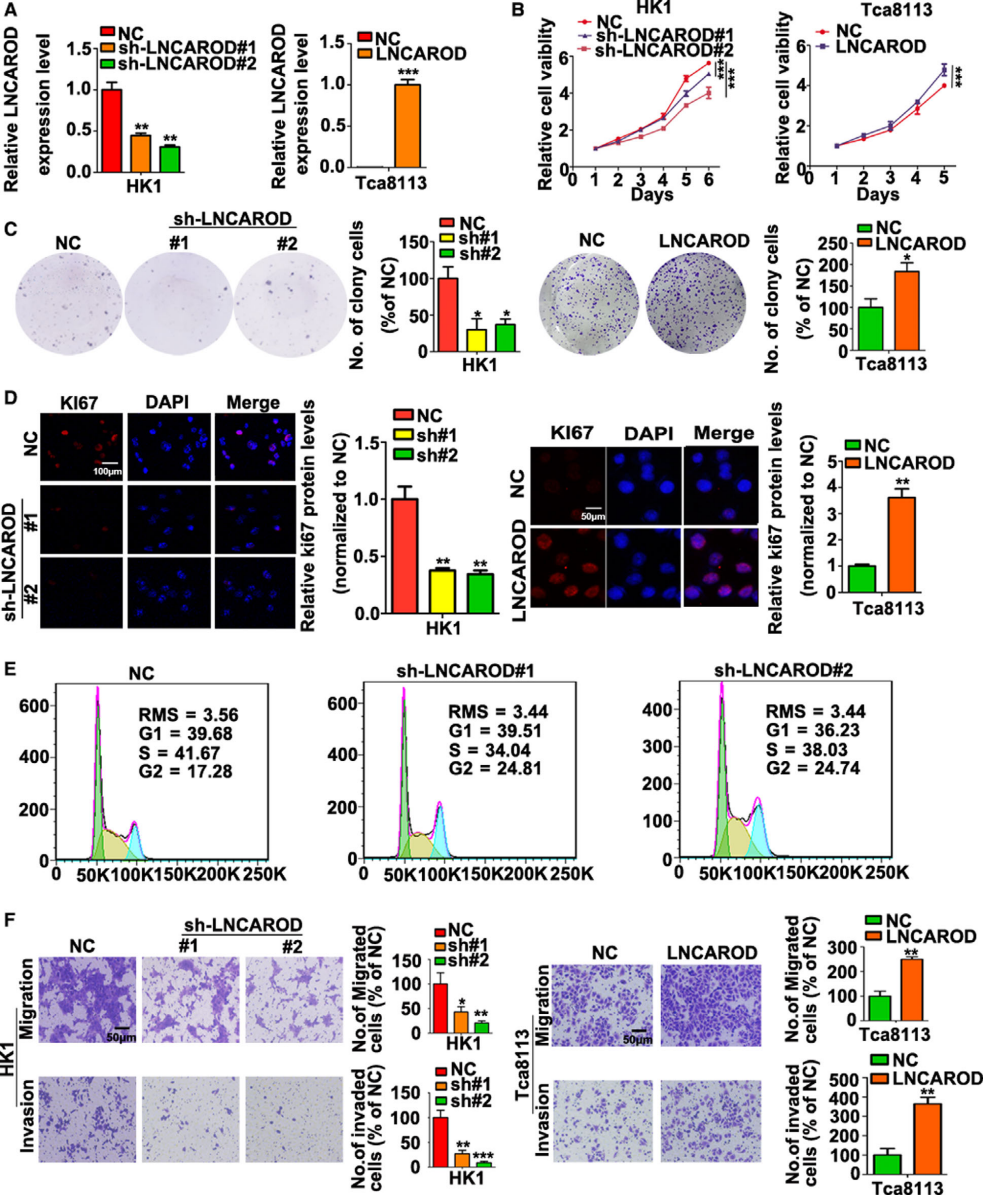
LNCAROD promotes growth, mobility, and invasiveness of HNSCC cells. (A) LNCAROD levels in stable silenced or stable transfected cells were determined by RT‐PCR assays (*n* = 3 per group. Data were analyzed using Student's *t*‐test). (B) CCK‐8 assays indicated that stable silencing LNCAROD suppressed HK1 cell proliferation, whereas overexpression of LNCAROD accelerated cell proliferation of Tca8113 cell (*n* = 5 per group. Data were analyzed using two‐way ANOVA). (C) Colony formation assays demonstrated that silencing LNCAROD enhanced colony formation ability in HK1 cell, whereas ectopic LNCAROD exerted opposite effect in Tca8113 cell (*n* = 3 per group. Data were analyzed using Student's *t*‐test). (D) Immunofluorescence assays indicated that loss of LNCAROD reduced Ki‐67^+^ cell frequency in HK1 cell, whereas overexpression of LNCAROD exerted opposite effect in Tca8113 cell (*n* = 3 per group. Data were analyzed using Student's *t*‐test). (E) Flow cytometry assays suggested stable silencing LNCAROD induced cell cycle arrested at G2/M phase in HK1 cell. (F) Migration and invasion assays performed by using transwell inserts suggested that high expression of LNCAROD promotes migration and invasiveness of HNSCC cells *in vitro* (*n* = 3 per group. Data were analyzed using Student's *t*‐test). All data are mean ± SD. *P < 0.05, **P < 0.01, ***P < 0.001.

The expression of LNCAROD in HK1 and FaDu cells were transiently inhibited by siRNAs or ASOs specifically targeted to LNCAROD. As shown in Fig. 3A, both siRNAs and ASOs effectively suppressed expression level of LNCAROD. CCK‐8 assay showed that inhibition of LNCAROD by either siRNAs or ASOs led to inhibition of cell proliferation in HK1 and FaDu cells (Fig. 3B). Moreover, transient silencing of LNCAROD in HK1 and FaDu cells impaired cell mobility and invasiveness *in vitro* (Fig. 3C). Then, LNCAROD expression in HK1 cells was stably silenced by shRNAs expressing lentivirus (Fig. 4A). Stable depletion of LNCAROD resulted in inhibition of cell proliferation in HK1 cell. Whereas forced expression of LNCAROD in Tca8113 cells exerted opposite effect (Fig. 4B). As revealed by colony formation assays, depletion of LNCAROD in HK1 cell effectively reduced the colony number. By contrast, forced expression of LNCAROD led to increase of colony number of Tca8113 cell (Fig. 4C). Immunofluorescence assay indicated the frequency of Ki‐67^+^ cells significantly decreased upon stable silencing LNCAROD in HK1 cell. However, forced expression of LNCAROD increased number of Ki67^+^ cells in Tca8113 cell (Fig. 4D). Cell cycle analysis demonstrated that stable silencing LNCAROD led to cell cycle arrest at G2/M phase in HK1 cell (Fig. 4E). Furthermore, inhibition of LNCAROD impaired cell mobility and invasiveness in HK1 cell. In contrast, forced expression of LNCAROD increased mobility and invasiveness in Tca8113 cell (Fig. 4F). Thus, our data indicated that LNCAROD exerts tumor promotive role in HNSCC cells *in vitro*.

### 3.4 LNCAROD forms a complex with YBX1 and HSPA1A proteins and prevents proteasomal degradation of YBX1 protein

**Fig. 5:**
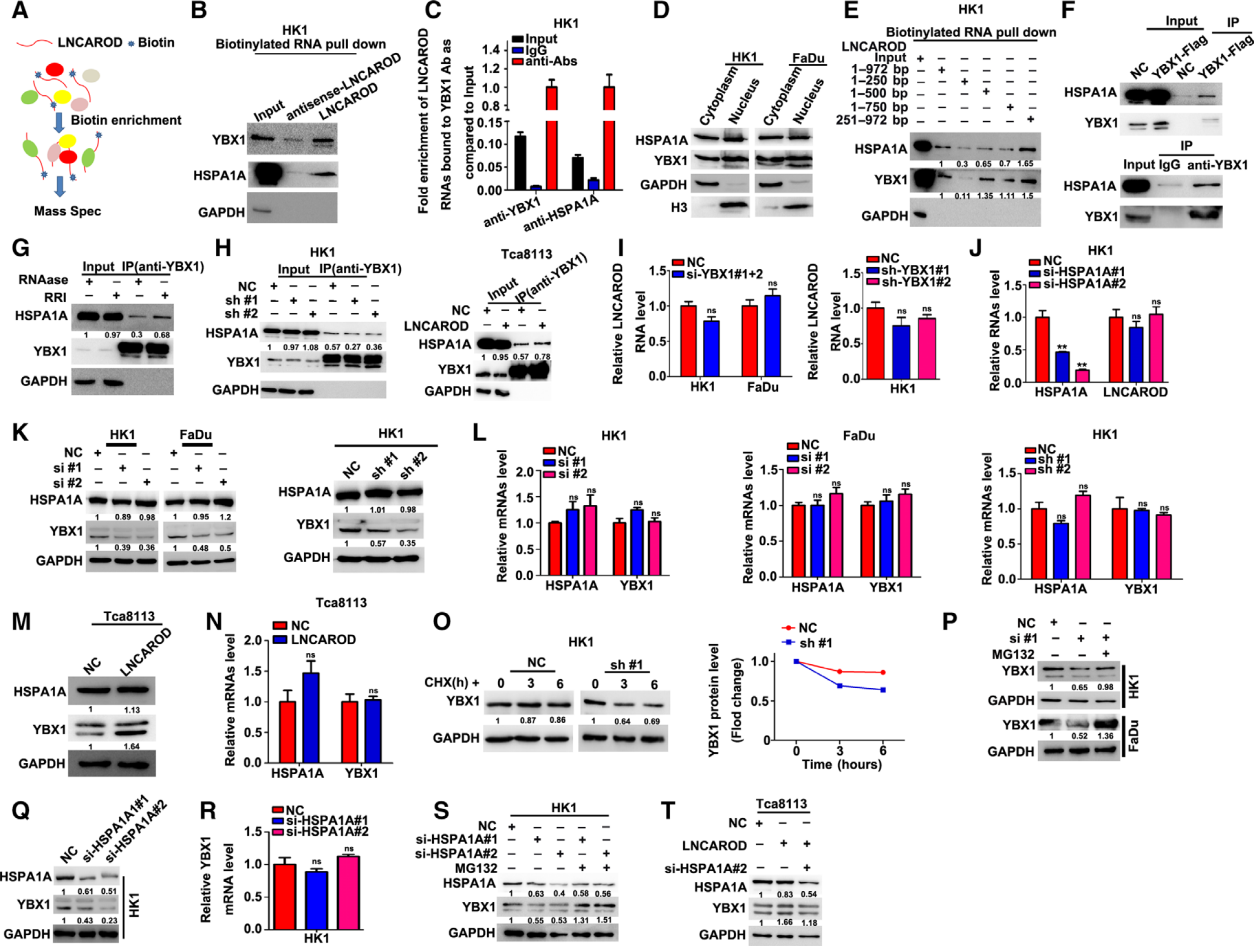
LNCAROD forms complex with HSPA1A and YBX1 and increases YBX1 protein stability in a HSPA1A‐dependent manner. (A) Schematic diagram of identification of LNCAROD binding proteins by RNA pull‐down coupled with mass spectrometry. (B) RNA pull‐down and western blot indicated that LNCAROD binds with HSPA1A and YBX1 proteins. (C) RIP‐qPCR assays demonstrated that HSPA1A and YBX1 bind with LNCAROD. (D) Subcellular fractionation and western blot assays indicated that YBX1 and HSPA1A proteins distribute in cytoplasm and nucleus in HK1 and FaDu cells. (E) Association of HSPA1A and YBX1 proteins with deletion fragments of LNCAROD were assessed by western blot. (F) Co‐IP assays suggested that both exogenous and endogenous YBX1 proteins were co‐immunoprecipitated with HSPA1A in HK1 cells. (G) Co‐IP assay revealed that RNase A treatment with cell lysate weakened YBX1‐HSPA1A association in HK1 cell. (H) YBX1‐HSPA1A association was assessed by Co‐IP assays in LNCAROD stably silenced HK1 cell and LNCAROD stably overexpressed Tca8113 cell. (I) RNA level of LNCAROD was measured in cells upon transient or stable loss of YBX1 (*n* = 3 per group). (J) RNA levels of HSPA1A and LNCAROD were measured by RT‐PCR upon transient silence of HSPA1A in HK1 cell (*n* = 3 per group). (K) HSPA1A and YBX1 protein levels in LNCAROD‐depleted cells were determined by western blot assays. (L) mRNAs levels of YBX1 and HSPAIA in HK1 and FaDu cells upon loss of LNCAROD were determined by RT‐qPCR assays (*n* = 3 per group). (M) HSPA1A and YBX1 protein levels were measured by western blot assays in Tca8113 cells with overexpression of LNCAROD. (N) HSPA1A and YBX1 mRNA levels in Tca8113 cell were measured by RT‐qPCR assays (*n* = 3 per group). (O) Pulse‐chase assay of YBX1 protein levels in HK1/sh‐LNCAROD cells treated with CHX. (P) MG132 treatment prevented reduction of YBX1 protein levels in HK1 and FaDu upon depletion of LNCAROD. (Q) YBX1 protein levels in HK1 cells transfected with HSPA1A specific siRNAs were measured by western blot assays. (R) YBX1 mRNA levels in HK1 cells transfected with HSPA1A specific siRNAs were measured by RT‐qPCR assays (*n* = 3 per group). (S) Western blot assays demonstrated that MG132 treatment prevented YBX1 protein degradation in HK1cell upon loss of HSPA1A. (T) YBX1 protein levels in Tca8113 cells were measured by western blot assays. All data are mean ± SD. Data were analyzed by using Student's *t*‐test. **P < 0.01.

We then sought to explore the molecular mechanisms underlying the oncogenic role of LNCAROD in HNSCC progression. A proteomic approach was employed to identify protein binding partners of *in vitro* biotinylated LNCAROD transcript in HK1 cell (Fig. 5A). Mass spectrometry analysis revealed that YBX1 and HSPA1A bind with LNCAROD. The binding between LNCAROD with YBX1 and HSPA1A was further validated by western blot following RNA pull‐down assays (Fig. 5B). Moreover, RIP assays demonstrated that LNCAROD RNA was precipitated with by anti‐YBX1 and anti‐HSPA1A in HK1 cell (Fig. 5C). Subcellular fractionation of HK1 and FaDu cells showed that YBX1 and HSPA1A proteins were distributed in cytoplasm and nucleus (Fig. 5D). Deletion mutant assays demonstrated LNCAROD binds with HSP1A1 through a region of its 3′ terminus (751–972 nt), whereas binds with YBX1 through its internal region (251–500 nt) (Fig. 5E). We also demonstrated that both exogenous and endogenous YBX1 protein co‐immunoprecipitated with HSPA1A protein in HK1 cell (Fig. 5F). However, RNase A pretreatment with the cell lysate significantly reduced YBX1‐HSPA1A association as compared to that pretreated with recombinant RNase inhibitor, suggesting a role of RNA involved (Fig. 5G). Furthermore, silencing LNCAROD in HK1 cells hindered the protein–protein interaction between YBX1 and HSPA1A, whereas overexpression of LNCAROD enhanced YBX1‐HSPA1A proteins interaction (Fig. 5H). Two specific siRNAs effectively repressed mRNA and protein level of YBX1 in HK1 cells (Fig. S2A). As shown in Fig. S2B,C, either transient or stable silencing effectively suppressed expression level of YBX1 in HK1 and FaDu cells. Silencing either YBX1 or HSPA1A in HK1 and FaDu cells exert little effect on the level of LNCAROD (Fig. 5I,J). However, either transient or stable inhibition of LNCAROD led to decrease of YBX1 protein level (Fig. 5K), without affecting YBX1 mRNA level (Fig. 5L). Unlike YBX1, both mRNA and protein level of HSPA1A remained unchanged upon loss of LNCAROD (Fig. 5K,L). In contrast, overexpression of LNCAROD led to upregulation of YBX1 protein level (Fig. 5M) without affecting its mRNA level (Fig. 5N). We further demonstrated that loss of LNCAROD shortened the half‐life of YBX1 protein (Fig. 5O), whereas proteasome inhibitor MG132 treatment partially rescued YBX1 protein upon silencing LNCAROD (Fig. 5P), suggesting loss of LNCAROD promotes proteasomal degradation of YBX1 protein. We then asked whether HSPA1A contributes to stabilization of YBX1 protein by LNCAROD in HNSCC cells. As expected, silencing HSPA1A in HK1 cell resulted in reduction of YBX1 protein level (Fig. 5Q), without affecting its mRNA level (Fig. 5R). MG132 treatment prevented reduction of YBX1 protein level in HK1 cells upon depletion of HSPA1A (Fig. 5S), indicating that HSPA1A inhibits proteasomal degradation of YBX1 protein. Furthermore, silencing HSPA1A led to reduction of YBX1 protein in LNCAROD‐overexpressing Tca8113 cell, suggesting that HSPA1A is required for LNCAROD‐mediated YBX1 protein stabilization (Fig. 5T). Thus, our data suggest that LNCAROD prevents proteasomal degradation of YBX1 protein through facilitating YBX1‐HSPA1A interaction.

### 3.5 LNCAROD promotes HNSCC cells malignant behaviors via maintaining YBX1 protein level

**Fig. 6:**
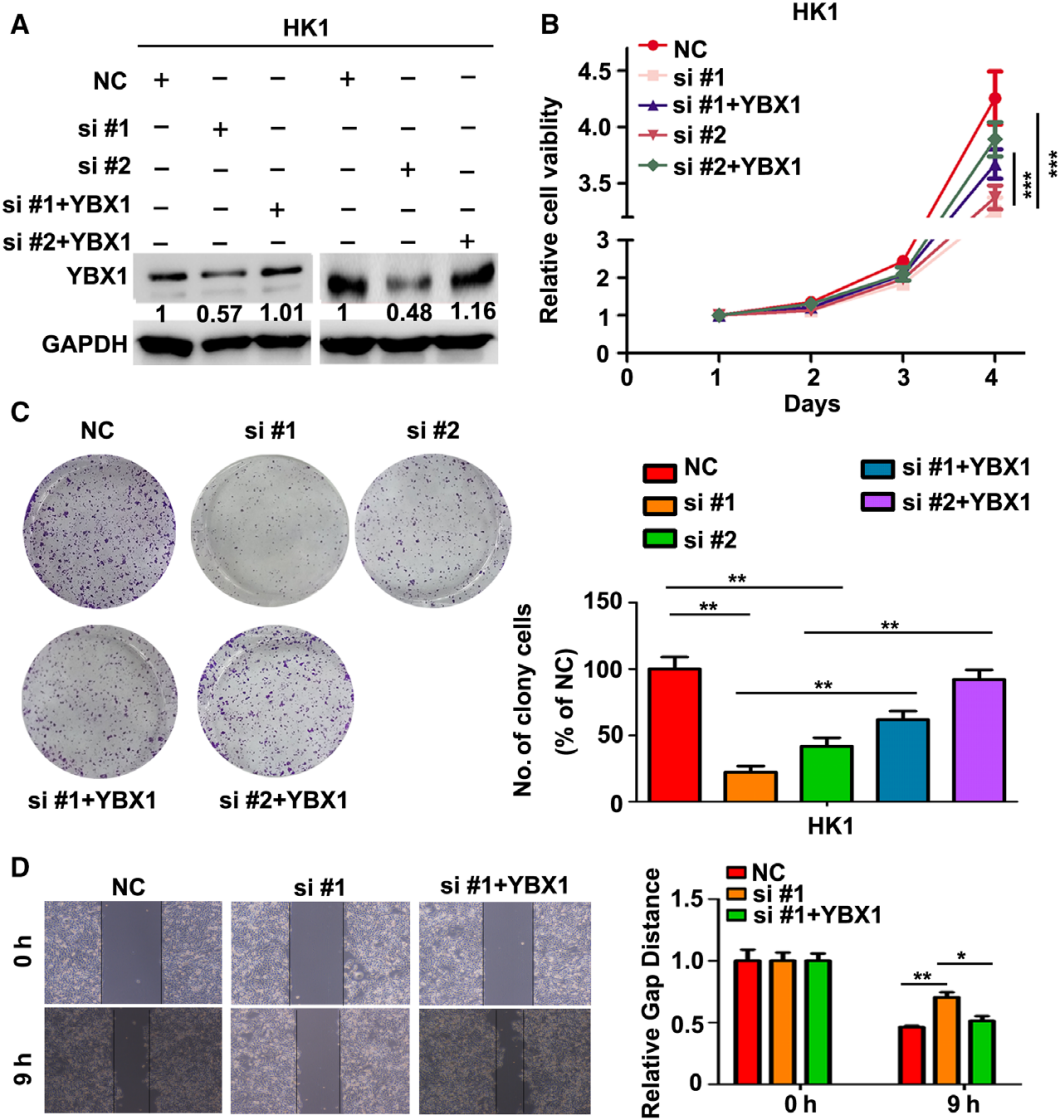
Re‐expression of YBX1 rescues aggressiveness of HNSCC cell upon silencing LNCAROD. (A) Protein level of YBX1 was measured by western blot assays. (B) Growth of HK1 cells were determined by CCK‐8 assays (*n* = 5 per group. Data were analyzed using two‐way ANOVA). (C) Cell survival ability of HK1 cells were measured by colony formation assays (*n* = 3 per group. Data were analyzed using Student's *t*‐test). (D) Cell migration was measured by wound healing assays (*n* = 3 per group. Data were analyzed using Student's *t*‐test). All data are mean ± SD. *P < 0.05, **P < 0.01, ***P < 0.001.

We then asked whether oncogenic effect of LNCAROD in HNSCC cells is mediated by YBX1. Re‐expression of Flag‐tagged YBX1 resulted in increase of YBX1 protein level in LNCAROD‐silenced HK1 cells (Fig. 6A; Fig. S3). Functionally, re‐expression of Flag‐YBX1 rescued cell proliferation and colony formation ability in LNCAROD‐silenced cells (Fig. 6B,C). Moreover, wound healing assays showed re‐expression of YBX1 in LNCAROD‐silenced cells led to restoration of cell mobility (Fig. 6D). Thus, our data clearly demonstrated that LNCAROD promotes HNSCC cells malignant behaviors via stabilization of YBX1 protein.

### 3.6 LNCAROD enhances tumor formation *in vivo*

**Fig. 7:**
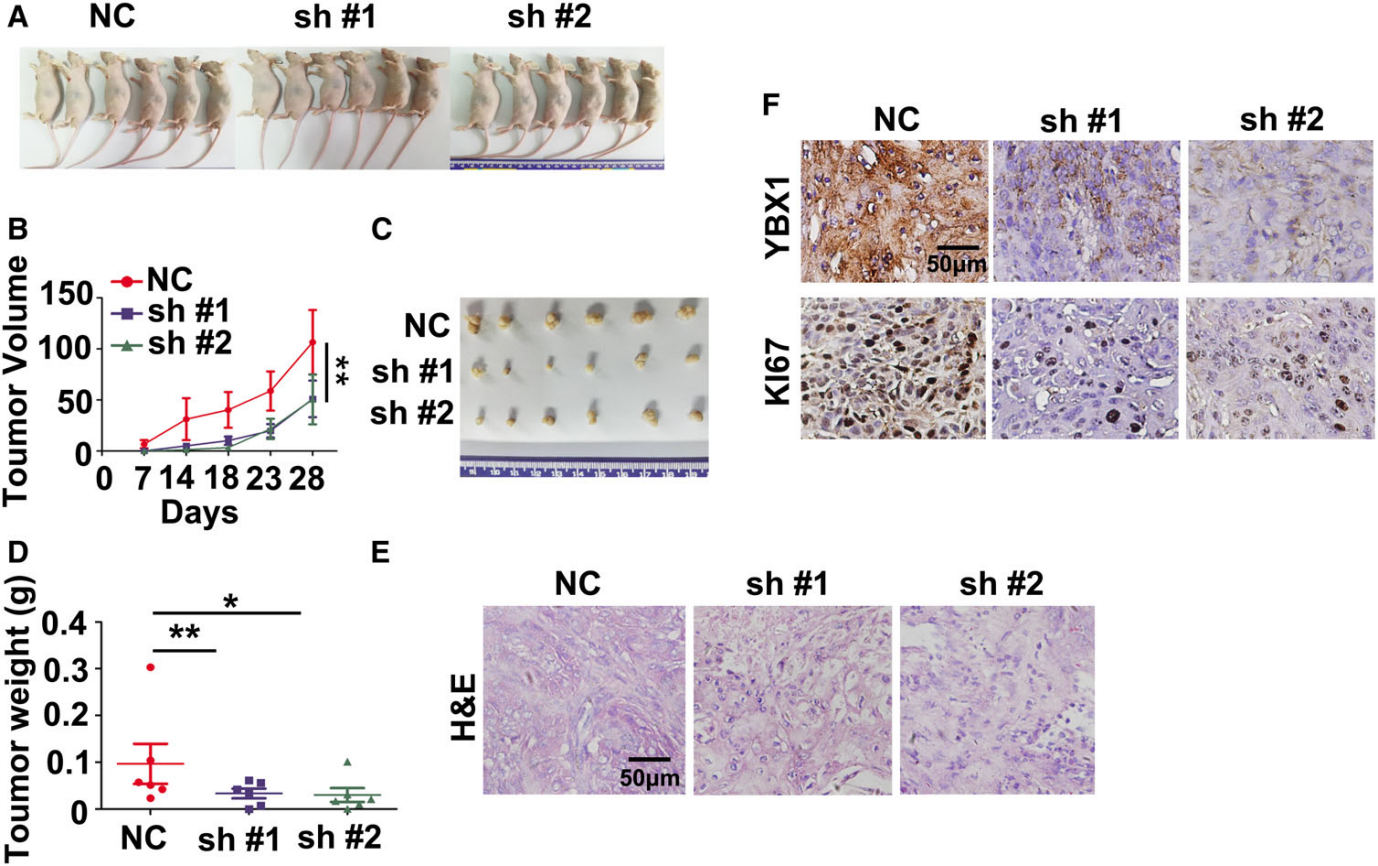
LNCAROD promotes tumor formation *in vivo*. (A) Gross view of nude mice bearing xenograft tumors. (B) Growth curve of xenograft tumors from LNCAROD‐silenced or control HK1 cells (*n* = 6 per group. Data were analyzed using two‐way ANOVA). (C) Macroscopic view of xenograft tumors from LNCAROD‐silenced or control HK1 cells. (D) Measurement of the weight of xenograft tumors (*n* = 6 per group. Data were analyzed using Student's *t*‐test). (E) H&E staining of xenograft tumors derived from LNCAROD‐silenced or control HK1 cells. (F) Immunohistochemical staining of YBX1 and Ki‐67 proteins in xenograft tumors. All data are mean ± SD. *P < 0.05, **P < 0.01.

We then asked whether high expression of LNCAROD enhances tumorigenicity of HNSCC cells *in vivo*. Xenograft tumor growth assays showed that loss of LNCAROD in HK1 cells delayed xenograft formation in nude mice (Fig. 7A,B). The volumes of xenograft tumors formed by two LNCAROD targeted shRNAs expressing cells were much smaller than that of vector control cell (Fig. 7C,D). Hematoxylin and eosin (H&E) staining showed that xenograft tumors from LNCAROD lower expressing cells exhibited a decreased nucleus‐to‐cytoplasm ratio, reduced nuclear atypia (Fig. 7E), suggesting reduced aggressiveness followed by inhibition of LNCAROD. Immunohistochemical staining showed there are less Ki67^+^ cells in tumors from sh‐LNCAROD/HK1 cells (Fig. 7F). Furthermore, the protein level of YBX1 was remarkably reduced upon silencing LNCAROD. These data indicate that LNCAROD acts as a *bona fide* oncogene in HNSCC.

## 4 Discussion

**Fig. 8:**
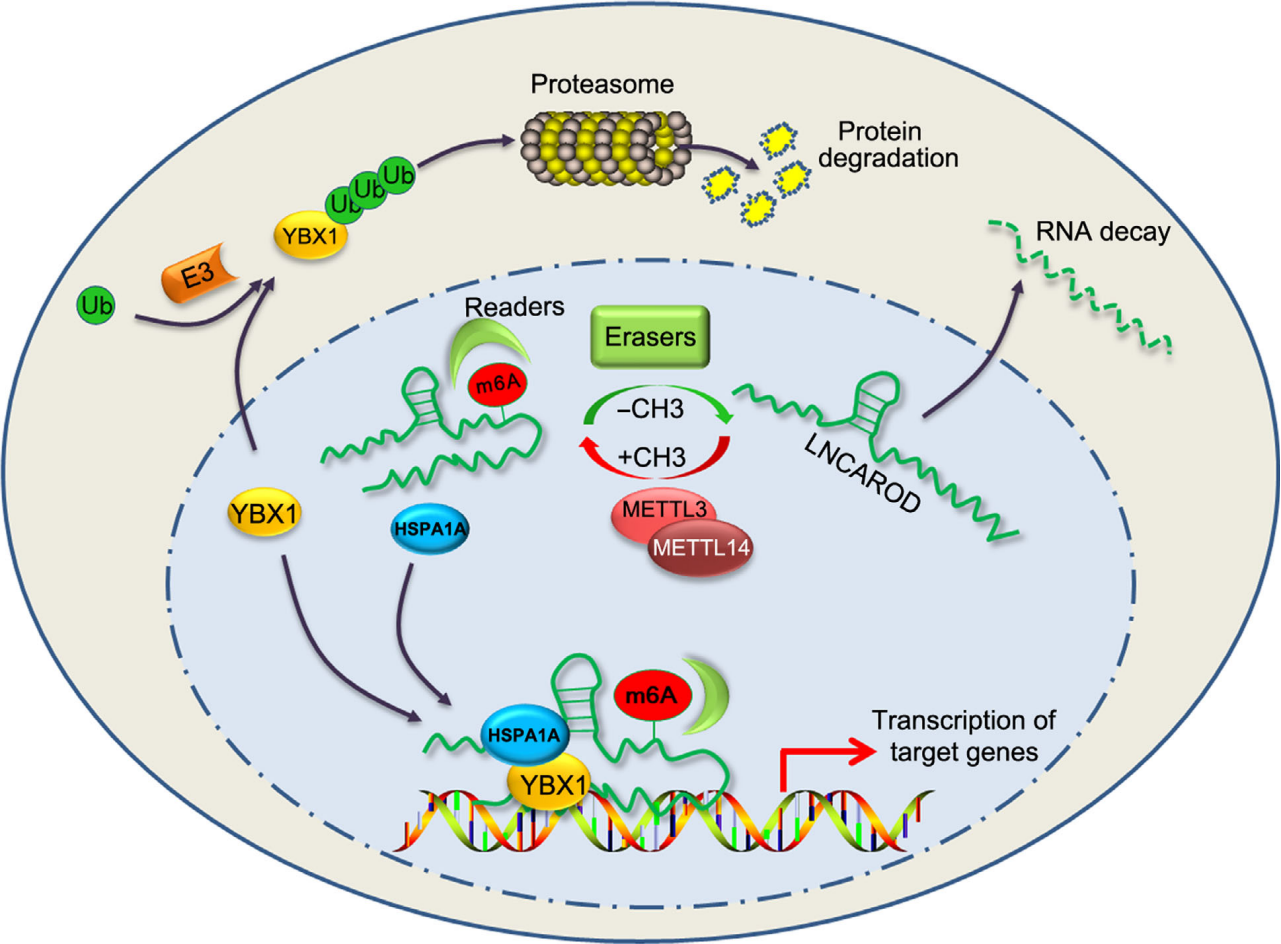
Schematic diagram of role of LNCAROD in HNSCC. METTL3‐ and METTL14‐mediated m6A methylation on LNCAROD extends the half‐life of LNCAROD. LNCAROD is mainly distributed in nucleus and links YBX1 and HSPA1A proteins together through binding to these two proteins, preventing proteasomal degradation of onogenic YBX1 protein in a HSPA1A‐dependent manner.

In this study, we uncovered the oncogenic role of LNCAROD in HNSCC development. Overexpression of LNCAROD is associated with advanced T stage and predicts poor overall prognosis in HNSCC. LNCAROD is primarily distributed in nucleus and is regulated by METT3‐ and METTL14‐mediated m6A methylation in HNSCC. LNCAROD promotes the aggressiveness of HNSCC cells through facilitating protein–protein interaction between YBX1 and HSPA1A and thus stabilizing YBX1 protein (Fig. 8).

Growing evidences show that lncRNAs play an essential role in multiple stages of cancer development, including initiation and progression (Xie *et al.*, 2019). We demonstrated that LNCAROD is overexpressed in HNSCC tissues and cell lines, which are in consistent with a previous publication that LNCAROD (also named as lnc‐MBL2‐4) is overexpressed in TSCC (Gao *et al.*, 2014). High expression of LNCAROD associates with advanced T stage and unfavorable prognosis in HNSCC patients. To date, little is known about the mechanisms underlying dysregulation of LNCAROD during HNSCC development. In this study, we demonstrated that LNCAROD is m6A‐modified in HNSCC cells. Both METTL3 and METTL14 seem to be responsible for marking LNCAROD with *N*6‐methyladenosine, because silencing either METTL3 or METTL14 led to reduction of m6A modification on LNCAROD. Similar to protein‐coding genes, we demonstrated that m6A modification increased the half‐life of LNCAROD in HNSCC cells, suggesting m6A marks of LNCAROD mediated by RNA m6A methyltransferases might account for high expression of LNCAROD in HNSCC. Though silencing either METTL3 or METTL14 in HK1 cell led to reduction of LNCAROD. Only the level of METTL3, nor the METTL14, is positively associated with the level of LNCAROD in HNSCC samples. This might be explained that gene regulation is more complex in tumor tissues than in propagated cell lines. Dysregulation of m6A methylation is intensively involved in squamous cell carcinoma progression. This hypothesis is supported by an observation that METTL3 is elevated and plays an oncogenic role in a variety of squamous cell carcinoma from various organs (Zhao and Cui, 2019; Zhou *et al.*, 2019). Recently, it has been shown that METTL3‐mediated m6A marks contribute to high expression of lncRNAs in glioma stem‐like cells (Visvanathan *et al.*, 2019). Thus, our data along with other observations unveiled a critical role of m6A modification on the fate and function of lncRNAs. The fate of m6A modified RNAs is largely dependent on recognition by different m6A readers (Patil *et al.*, 2018). Recognition by YTHDFs promotes degradation of *N*(6)‐methyladenosine‐modified RNA (Wang *et al.*, 2014), whereas recognition of *N*(6)‐methyladenosine by IGF2BPs increases the stability of mRNAs (Huang *et al.*, 2018). We speculated that m6A modification probably protects LNCAROD from degradation through enhancing its recognition by IGF2BPs family members. However, this assumption needs more study to decipher whether it is a universal law or not.

Before our study, LNCAROD was demonstrated to activate DKK1 transcription in breast cancer MCF‐7 cell (Ntini *et al.*, 2018). However, silencing LNCAROD did not alter DKK1 level in our systems (data not shown). This discrepancy might be explained that LNCAROD exerts different roles in various cell contexts. YBX1 is a multifunctional DNA‐/RNA‐binding protein. It consists of three domains: a cold shock protein (CSD) domain, an A/P domain, and a long C‐terminal domain (Suresh *et al.*, 2018). Apart from mRNAs, growing evidence revealed that YBX1 is intensively involved in regulating noncoding RNA expression (Suresh *et al.*, 2018). It has been shown that binding with RNAs increases YBX1 protein stability (Dimartino *et al.*, 2018; Su *et al.*, 2018). We demonstrated that LNCAROD binds with YBX1 or HSPA1A through different fragments, 251–500 nt for YBX1 and 751–972 nt for HSPA1A, respectively, making it as a scaffold for YBX1‐HSPA1A interaction. Though HSPA1A protein was co‐immunoprecipitated with YBX1 protein, its interaction was impeded upon loss of LNCAROD and even dramatically abolished by pretreatment of cell lysate with RNase A, indicating a role of RNAs involved. The protein–protein interaction between YBX1 and HSPA1A is important for stabilization of YBX1 protein, since depletion of HSPA1A reduced YBX1 protein level in LNCAROD high expressing cells. HSPA1A is a member of Hsp70 chaperones which are implicated in controlling proteins stability through regulation of conformation of the targeted proteins. In a previous study, HSPA1A was found to protect ZNF198‐FGFR1 fusion protein from proteasomal degradation (Kasyapa *et al.*, 2007). We assumed that binding to LNCAROD makes HSPA1A spatially close to YBX1 protein bound with LNCAROD and induce conformation change of YBX1 protein, preventing recognition of YBX1 by E3‐ubiquitin ligase (Chibi *et al.*, 2008; Lutz *et al.*, 2006) (Fig. 8). Given that pretreatment with RNase A more effectively impaired YBX1‐HSPA1A interaction than single silencing LNCAROD, we speculate that there are other RNA molecules involved in YBX1‐HSPA1A interaction. It has been shown that several lncRNAs, including MIR22HG (Su *et al.*, 2018), lnc‐31 (Dimartino *et al.*, 2018), interact with YBX1 protein and increase its stability. Also, Hsp70 possesses RNA‐binding activities in a protein chaperone functions independent manner (Kishor *et al.*, 2017). Therefore, it is highly probable that the interaction between YBX1 and HSPA1A could be mediated by various RNA molecules in different cell contexts.

## 5 Conclusions

In this study, we unveiled the tumor promotive function of LNCAROD in HNSCC development. We provide evidence that m6A methylation mediated by METTL3 and METTL14 contributes to increased stability of LNCAROD. Overexpression of LNCAROD promotes malignant development of HNSCC through facilitating YBX1–HSPA1A interaction, thus enhancing YBX1 protein stability. Our study shed light on the mechanisms of lncRNAs in HNSCC development.

## Conflict of interest

The authors declare no conflict of interest.

## Author contributions

BX, MY, and YB designed the study. YB, PT, JC, YZ, JL, MH, and YM performed the majority of the experiments. YT, YB, and BX analyzed the data. MY and BX wrote the manuscript. ZZ, XLL, WX, GL, and XYL revised and corrected the manuscript.

## Supporting information


**Fig. S1.** Schematic diagram of strategy to screen differentially expressed lncRNAs in HNSCC.

**Fig. S2.** Silencing YBX1 expression in tumor cells.

**Fig. S3.** Exogenous Flag‐YBX1 expression in HK1 cell.

**Table S1.** Sequences of siRNAs and ASOs used in this study.

**Table S2.** Sequences of specific primers for lncRNAs and mRNAs used in this study.

**Appendix S1.** The differentially expressed lncRNAs between HNSCC and normal tissues.

Supplementary material

## Data Availability

All experimental data during this research are included in the published article and its supplementary files. The datasets and materials in this study are available on reasonable request from corresponding authors.

## References

[mol212676-bib-0001] Barbieri I , Tzelepis K , Pandolfini L , Shi J , Millan‐Zambrano G , Robson SC , Aspris D , Migliori V , Bannister AJ , Han N *et al* (2017) Promoter‐bound METTL3 maintains myeloid leukaemia by m(6)A‐dependent translation control. Nature 552, 126–131. 29186125 10.1038/nature24678 PMC6217924

[mol212676-bib-0002] Blot WJ , McLaughlin JK , Winn DM , Austin DF , Greenberg RS , Preston‐Martin S , Bernstein L , Schoenberg JB , Stemhagen A and Fraumeni JF Jr (1988) Smoking and drinking in relation to oral and pharyngeal cancer. Can Res 48, 3282–3287. 3365707

[mol212676-bib-0003] Bray F , Ferlay J , Soerjomataram I , Siegel RL , Torre LA and Jemal A (2018) Global cancer statistics 2018: GLOBOCAN estimates of incidence and mortality worldwide for 36 cancers in 185 countries. CA Cancer J Clin 68, 394–424. 30207593 10.3322/caac.21492

[mol212676-bib-0004] Chaturvedi AK , Engels EA , Pfeiffer RM , Hernandez BY , Xiao W , Kim E , Jiang B , Goodman MT , Sibug‐Saber M , Cozen W *et al* (2011) Human papillomavirus and rising oropharyngeal cancer incidence in the United States. J Clin Oncol 29, 4294–4301. 21969503 10.1200/JCO.2011.36.4596 PMC3221528

[mol212676-bib-0005] Chen XY , Zhang J and Zhu JS (2019) The role of m(6)A RNA methylation in human cancer. Mol Cancer 18, 103. 31142332 10.1186/s12943-019-1033-z PMC6540575

[mol212676-bib-0006] Cheung ST , Huang DP , Hui AB , Lo KW , Ko CW , Tsang YS , Wong N , Whitney BM and Lee JC (1999) Nasopharyngeal carcinoma cell line (C666–1) consistently harbouring Epstein‐Barr virus. Int J Cancer 83, 121–126. 10449618 10.1002/(sici)1097-0215(19990924)83:1<121::aid-ijc21>3.0.co;2-f

[mol212676-bib-0007] Chibi M , Meyer M , Skepu A , Rees DJG , Moolman‐Smook JC and Pugh DJR (2008) RBBP6 interacts with multifunctional protein YB‐1 through Its RING finger domain, leading to ubiquitination and proteosomal degradation of YB‐1. J Mol Biol 384, 908–916. 18851979 10.1016/j.jmb.2008.09.060

[mol212676-bib-0008] Coker H , Wei G and Brockdorff N (2019) m6A modification of non‐coding RNA and the control of mammalian gene expression. Biochim Biophys Acta Gene Regul Mech 1862, 310–318. 30550772 10.1016/j.bbagrm.2018.12.002

[mol212676-bib-0009] Cramer JD , Burtness B , Le QT and Ferris RL (2019) The changing therapeutic landscape of head and neck cancer. Nat Rev Clin Oncol 16, 669–683. 31189965 10.1038/s41571-019-0227-z

[mol212676-bib-0010] Dimartino D , Colantoni A , Ballarino M , Martone J , Mariani D , Danner J , Bruckmann A , Meister G , Morlando M and Bozzoni I (2018) The long non‐coding RNA lnc‐31 interacts with Rock1 mRNA and mediates its YB‐1‐dependent translation. Cell Rep 23, 733–740. 29669280 10.1016/j.celrep.2018.03.101 PMC5917449

[mol212676-bib-0011] Fan C , Tang Y , Wang J , Xiong F , Guo C , Wang Y , Zhang S , Gong Z , Wei F , Yang L *et al* (2017) Role of long non‐coding RNAs in glucose metabolism in cancer. Mol Cancer 16, 130. 28738810 10.1186/s12943-017-0699-3 PMC5525357

[mol212676-bib-0012] Gao W , Chan JY and Wong TS (2014) Long non‐coding RNA deregulation in tongue squamous cell carcinoma. Biomed Res Int 2014, 405860. 25045670 10.1155/2014/405860 PMC4090519

[mol212676-bib-0013] Gao X , Zhang S , Chen Y , Wen X , Chen M , Wang S and Zhang Y (2019) Development of a novel six‐long noncoding RNA signature predicting survival of patients with bladder urothelial carcinoma. J Cell Biochem 120, 19796–19809. 31338862 10.1002/jcb.29285

[mol212676-bib-0014] Hegde GV , de la Cruz C , Giltnane JM , Crocker L , Venkatanarayan A , Schaefer G , Dunlap D , Hoeck JD , Piskol R , Gnad F *et al* (2019) NRG1 is a critical regulator of differentiation in TP63‐driven squamous cell carcinoma. eLife 8, e46551. 31144617 10.7554/eLife.46551 PMC6606022

[mol212676-bib-0015] Huang DP , Ho JH , Poon YF , Chew EC , Saw D , Lui M , Li CL , Mak LS , Lai SH and Lau WH (1980) Establishment of a cell line (NPC/HK1) from a differentiated squamous carcinoma of the nasopharynx. Int J Cancer 26, 127–132. 6259064 10.1002/ijc.2910260202

[mol212676-bib-0016] Huang H , Weng H , Sun W , Qin X , Shi H , Wu H , Zhao BS , Mesquita A , Liu C , Yuan CL *et al* (2018) Recognition of RNA N(6)‐methyladenosine by IGF2BP proteins enhances mRNA stability and translation. Nat Cell Biol 20, 285–295. 29476152 10.1038/s41556-018-0045-z PMC5826585

[mol212676-bib-0017] Kasyapa CS , Kunapuli P and Cowell JK (2007) HSPA1 a is an important regulator of the stability and function of ZNF198 and its oncogenic derivative, ZNJF198‐FGFR1. J Cell Biochem 102, 1308–1317. 17471537 10.1002/jcb.21362

[mol212676-bib-0018] Kishor A , White EJF , Matsangos AE , Yan Z , Tandukar B and Wilson GM (2017) Hsp70's RNA‐binding and mRNA‐stabilizing activities are independent of its protein chaperone functions. J Biol Chem 292, 14122–14133. 28679534 10.1074/jbc.M117.785394 PMC5572911

[mol212676-bib-0019] Lan Q , Liu PY , Haase J , Bell JL , Huttelmaier S and Liu T (2019) The critical role of RNA m(6)A methylation in cancer. Can Res 79, 1285–1292. 10.1158/0008-5472.CAN-18-2965 30894375

[mol212676-bib-0020] Li J , Wang W , Chen S , Cai J , Ban Y , Peng Q , Zhou Y , Zeng Z , Li X , Xiong W *et al* (2019) FOXA1 reprograms the TGF‐beta‐stimulated transcriptional program from a metastasis promoter to a tumor suppressor in nasopharyngeal carcinoma. Cancer Lett 442, 1–14. 30392786 10.1016/j.canlet.2018.10.036

[mol212676-bib-0021] Livak KJ and Schmittgen TD (2001) Analysis of relative gene expression data using real‐time quantitative PCR and the 2(‐Delta Delta C(T)) method. Methods 25, 402–408. 11846609 10.1006/meth.2001.1262

[mol212676-bib-0022] Lutz M , Wempe F , Bahr I , Zopf D and von Melchner H (2006) Proteasomal degradation of the multifunctional regulator YB‐1 is mediated by an F‐Box protein induced during programmed cell death. Febs Lett 580, 3921–3930. 16797541 10.1016/j.febslet.2006.06.023

[mol212676-bib-0023] Maier H , Dietz A , Gewelke U , Heller WD and Weidauer H (1992) Tobacco and alcohol and the risk of head and neck cancer. Clin Investig 70, 320–327. 10.1007/BF00184668 1521046

[mol212676-bib-0024] Noh JH , Kim KM , McClusky WG , Abdelmohsen K and Gorospe M (2018) Cytoplasmic functions of long noncoding RNAs. Wiley Interdiscip Rev RNA 9, e1471. 29516680 10.1002/wrna.1471 PMC5963534

[mol212676-bib-0025] Ntini E , Louloupi A , Liz J , Muino JM , Marsico A and Orom UAV (2018) Long ncRNA A‐ROD activates its target gene DKK1 at its release from chromatin. Nat Commun 9, 1636. 29691407 10.1038/s41467-018-04100-3 PMC5915440

[mol212676-bib-0026] Patil DP , Pickering BF and Jaffrey SR (2018) Reading m(6)A in the transcriptome: m(6)A‐binding proteins. Trends Cell Biol 28, 113–127. 29103884 10.1016/j.tcb.2017.10.001 PMC5794650

[mol212676-bib-0027] Raj A and Rinn JL (2019) Illuminating genomic dark matter with RNA imaging. Cold Spring Harb Perspect Biol 11, a032094. 31043413 10.1101/cshperspect.a032094 PMC6496349

[mol212676-bib-0028] Su W , Feng S , Chen X , Yang X , Mao R , Guo C , Wang Z , Thomas DG , Lin J , Reddy RM *et al* (2018) Silencing of long noncoding RNA MIR22HG triggers cell survival/death signaling via oncogenes YBX1, MET, and p21 in lung cancer. Can Res 78, 3207–3219. 10.1158/0008-5472.CAN-18-0222 PMC6004254 29669758

[mol212676-bib-0029] Suresh PS , Tsutsumi R and Venkatesh T (2018) YBX1 at the crossroads of non‐coding transcriptome, exosomal, and cytoplasmic granular signaling. Eur J Cell Biol 97, 163–167. 29478751 10.1016/j.ejcb.2018.02.003

[mol212676-bib-0030] Tang Y , He Y , Zhang P , Wang J , Fan C , Yang L , Xiong F , Zhang S , Gong Z , Nie S *et al* (2018) LncRNAs regulate the cytoskeleton and related Rho/ROCK signaling in cancer metastasis. Mol Cancer 17, 77. 29618386 10.1186/s12943-018-0825-x PMC5885413

[mol212676-bib-0031] Tang Y , Wang J , Lian Y , Fan C , Zhang P , Wu Y , Li X , Xiong F , Li X , Li G *et al* (2017) Linking long non‐coding RNAs and SWI/SNF complexes to chromatin remodeling in cancer. Mol Cancer 16, 42. 28212646 10.1186/s12943-017-0612-0 PMC5316185

[mol212676-bib-0032] Tsao SW , Wang X , Liu Y , Cheung YC , Feng H , Zheng Z , Wong N , Yuen PW , Lo AK , Wong YC *et al* (2002) Establishment of two immortalized nasopharyngeal epithelial cell lines using SV40 large T and HPV16E6/E7 viral oncogenes. Biochem Biophys Acta 1590, 150–158. 12063178 10.1016/s0167-4889(02)00208-2

[mol212676-bib-0033] Visvanathan A , Patil V , Abdulla S , Hoheisel JD and Somasundaram K (2019) N‐6‐methyladenosine landscape of glioma stem‐like cells: METTL3 is essential for the expression of actively transcribed genes and sustenance of the oncogenic signaling. Genes (Basel) 10, E141. 30781903 10.3390/genes10020141 PMC6410051

[mol212676-bib-0034] Visvanathan A , Patil V , Arora A , Hegde AS , Arivazhagan A , Santosh V and Somasundaram K (2018) Essential role of METTL3‐mediated m(6)A modification in glioma stem‐like cells maintenance and radioresistance. Oncogene 37, 522–533. 28991227 10.1038/onc.2017.351

[mol212676-bib-0035] Vu LP , Pickering BF , Cheng Y , Zaccara S , Nguyen D , Minuesa G , Chou T , Chow A , Saletore Y , MacKay M *et al* (2017) The N(6)‐methyladenosine (m(6)A)‐forming enzyme METTL3 controls myeloid differentiation of normal hematopoietic and leukemia cells. Nat Med 23, 1369–1376. 28920958 10.1038/nm.4416 PMC5677536

[mol212676-bib-0036] Wang X , Lu Z , Gomez A , Hon GC , Yue Y , Han D , Fu Y , Parisien M , Dai Q , Jia G *et al* (2014) N6‐methyladenosine‐dependent regulation of messenger RNA stability. Nature 505, 117–120. 24284625 10.1038/nature12730 PMC3877715

[mol212676-bib-0037] Weng H , Huang H , Wu H , Qin X , Zhao BS , Dong L , Shi H , Skibbe J , Shen C , Hu C *et al* (2018) METTL14 inhibits hematopoietic stem/progenitor differentiation and promotes leukemogenesis via mRNA m(6)A modification. Cell Stem Cell 22, 191–205.e9. 29290617 10.1016/j.stem.2017.11.016 PMC5860916

[mol212676-bib-0038] Xie Y , Dang W , Zhang S , Yue W , Yang L , Zhai X , Yan Q and Lu J (2019) The role of exosomal noncoding RNAs in cancer. Mol Cancer 18, 37. 30849983 10.1186/s12943-019-0984-4 PMC6408816

[mol212676-bib-0039] Yamamoto T and Saitoh N (2019) Non‐coding RNAs and chromatin domains. Curr Opin Cell Biol 58, 26–33. 30682683 10.1016/j.ceb.2018.12.005

[mol212676-bib-0040] Yao RW , Wang Y and Chen LL (2019) Cellular functions of long noncoding RNAs. Nat Cell Biol 21, 542–551. 31048766 10.1038/s41556-019-0311-8

[mol212676-bib-0041] Zhao X and Cui L (2019) Development and validation of a m(6)A RNA methylation regulators‐based signature for predicting the prognosis of head and neck squamous cell carcinoma. Am J Cancer Res 9, 2156–2169. 31720080 PMC6834477

[mol212676-bib-0042] Zhou R , Gao Y , Lv D , Wang C , Wang D and Li Q (2019) METTL3 mediated m(6)A modification plays an oncogenic role in cutaneous squamous cell carcinoma by regulating DeltaNp63. Biochem Biophys Res Comm 515, 310–317. 31153635 10.1016/j.bbrc.2019.05.155

