# Supplementary figures and images for: LNCAROD is stabilized by m6A methylation and promotes cancer progression via forming a ternary complex with HSPA1A and YBX1 in head and neck squamous cell carcinoma
Source: Mol Oncol. 2020 Apr 13;14(6):1282–96. doi: 10.1002/1878-0261.12676 (PMC7266281; doi:10.1002/1878-0261.12676)

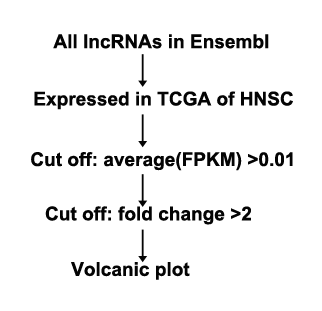

Supplement: Supplementary file 1 — Fig. S1. Schematic diagram of strategy to screen differentially expressed lncRNAs in HNSCC. [file MOL2-14-1282-s001.tif]

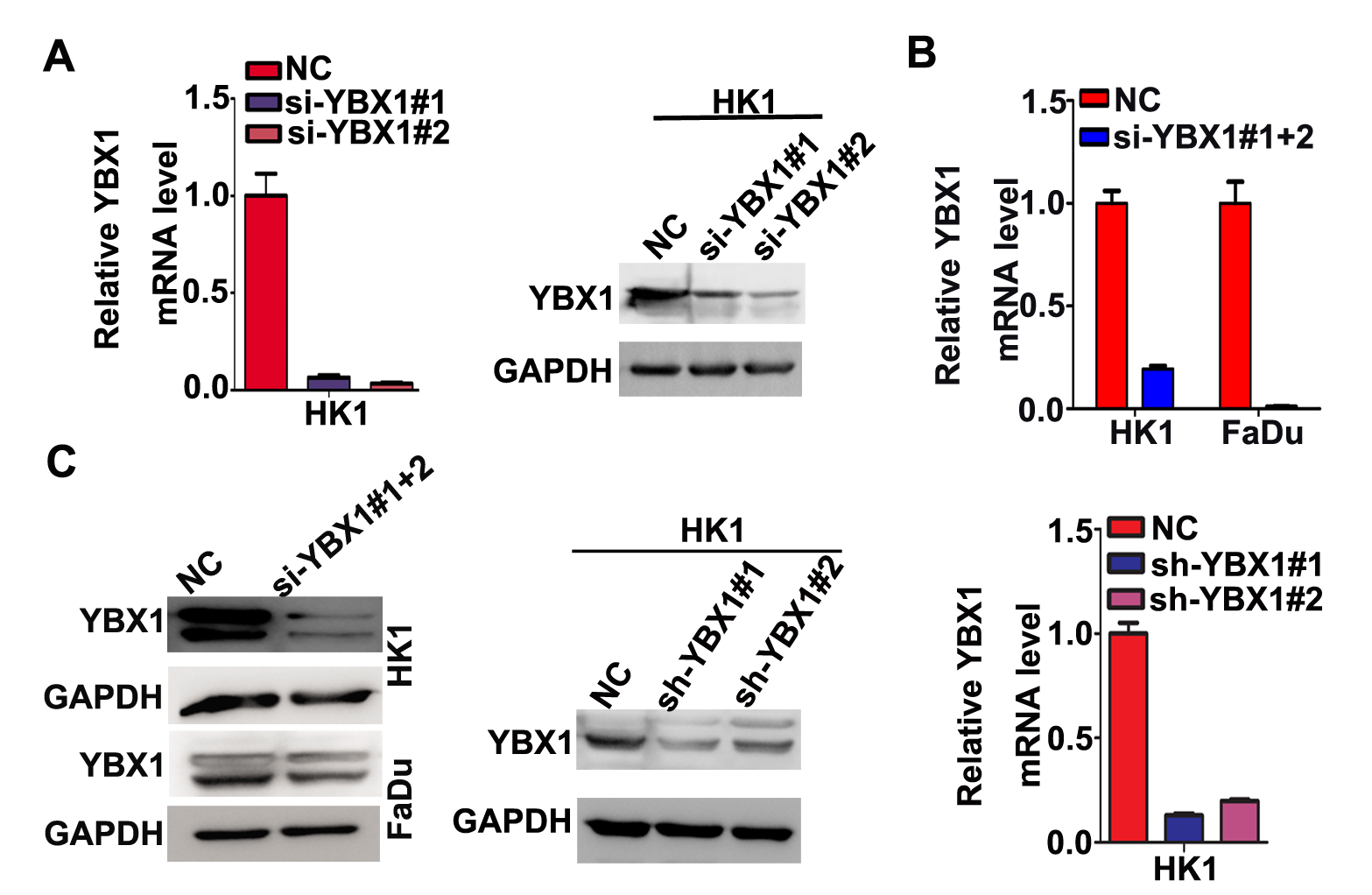

Supplement: Supplementary file 2 — Fig. S2. Silencing YBX1 expression in tumor cells. [file MOL2-14-1282-s002.tif]

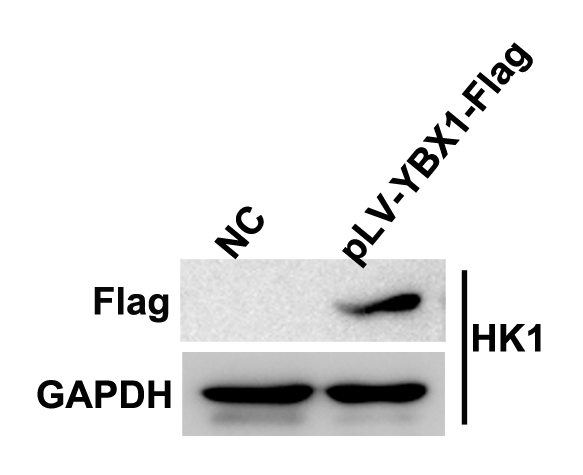

Supplement: Supplementary file 3 — Fig. S3. Exogenous Flag‐YBX1 expression in HK1 cell. [file MOL2-14-1282-s003.tif]
