# Supplementary material for: LNCAROD is stabilized by m6A methylation and promotes cancer progression via forming a ternary complex with HSPA1A and YBX1 in head and neck squamous cell carcinoma
Source: Mol Oncol. 2020 Apr 13;14(6):1282–96. doi: 10.1002/1878-0261.12676 (PMC7266281; doi:10.1002/1878-0261.12676)
Supplement: Supplementary file 4 — Table S1. Sequences of siRNAs and ASOs used in this study. [file MOL2-14-1282-s004.doc]

|  | Name | Sequence |
| --- | --- | --- |
| siRNA | Negative control (human) | UUCUCCGAACGUGUCACGUTT |
| siRNA | LNCAROD-1# (human) | TCGTGAAGATCTAATCGCAGTGAAA |
| siRNA | LNCAROD-2# (human) | GGGCTTGAGTCTCAACTCT |
| siRNA | YBX1-1# (human) | GACGGCAATGAAGAAGATAA |
| siRNA | YBX1-2# (human) | GTTCAATGTAAGGAACGGAT |
| siRNA | HSPA1A-1# (human) | CAGGTGATCAACGACGGAGAC |
| siRNA | HSPA1A-2# (human) | GAAGGACGAGTTTGAGCACAA |
| siRNA | METTL3 (human) | GCAAGUAUGUUCACUAUGATT |
| siRNA | METTL14 (human) | GUGCCGUGUUAAAUAGCAATT |
| ASO | LNCAROD-1#(human) | GTTGTTTGAAGCCGTGAGAC |
| ASO | LNCAROD-2#(human) | ACAACTCCAGTGGAACTCTG |

Table S1 Sequences of siRNAs and ASOs used in this study.
