# Supplementary material for: LNCAROD is stabilized by m6A methylation and promotes cancer progression via forming a ternary complex with HSPA1A and YBX1 in head and neck squamous cell carcinoma
Source: Mol Oncol. 2020 Apr 13;14(6):1282–96. doi: 10.1002/1878-0261.12676 (PMC7266281; doi:10.1002/1878-0261.12676)
Supplement: Supplementary file 5 — Table S2. Sequences of specific primers for lncRNAs and mRNAs used in this study. [file MOL2-14-1282-s005.doc]

| Name | Primer-F | Primer-R |
| --- | --- | --- |
| YBX1 | ACAAGAAGGTCATCGCAACG | AACTGGAACACCACCAGGAC |
| HSPA1A | AGCTGGAGCAGGTGTGTAAC | CAGCAATCTTGGAAAGGCCC |
| GAPDH | AACGGATTTGGTCGTATTGG | TTGATTTTGGAGGGATCTCG |
| METTL3 | TCTGGGGGTATGAACGGGTA | CTGGTTGAAGCCTTGGGGAT |
| METTL14 | GTAGCACAGACGGGGACTTC | GAGCCAGCCTGGTCGAATTG |
| LNCAROD | CCACAACGGCAACCAGTAAA | AGGCGTTCCACCTGCAAATA |
| LNCAROD-v1 | AGTGCATGCTCCATCACATC | GCTCCAGCATGCAGAGATAAAC |
| LNCAROD-v2 | GAGGGCCTGAGTCCTTGTAG | ATCCTCATCGCAGTAGCCTG |
| LNCAROD(1-972nt) | GCGCGGATCCATTCAAGATGATGTTAGA | CCGGAATTCTATCAGCAGACAGATATA |
| LNCAROD(1-250nt) | GCGCGGATCCATTCAAGATGATGTTAGA | ATATGAATTCGCTCCGTCTTCAGACT |
| LNCAROD(1-500nt) | GCGCGGATCCATTCAAGATGATGTTAGA | CGGAATTCTTCGAGGGCTACTGAAGA |
| LNCAROD(1-750nt) | GCGCGGATCCATTCAAGATGATGTTAGA | CCGGAATTCATGCAATCTCCTCTTCAT |
| LNCAROD(251-972nt) | ATATGGATCCAGCCCTGAGACACTCCATT | CCGGAATTCTATCAGCAGACAGATATA |
| pCDH-LNCAROD(1-972nt) | CGGCCGGAATTCATTCAAGATGATGTTAGA | GCGGATCCTATCAGCAGACAGATATA |

Table S2 Sequences of specific primers for lncRNAs and mRNAs used in this study.
