# Supplementary material for: LNCAROD is stabilized by m6A methylation and promotes cancer progression via forming a ternary complex with HSPA1A and YBX1 in head and neck squamous cell carcinoma
Source: Mol Oncol. 2020 Apr 13;14(6):1282–96. doi: 10.1002/1878-0261.12676 (PMC7266281; doi:10.1002/1878-0261.12676)
Supplement: Supplementary file 7 — Supplementary material [file MOL2-14-1282-s007.docx]

**Figure S1 Schematic diagram of strategy to screen differentially expressed lncRNAs in HNSCC**

LncRNAs list was downloaded from Ensemble database, and RNA-seq data of HNSCC samples were downloaded from TCGA database. Then the differentially expressed lncRNAs were assessed by using R software with a criteria of fold change > 2, *P*<0.05.

**Figure S2 Silencing YBX1 expression in tumor cells.**

A, YBX1 mRNA and protein levels in cells transfected siRNAs were measured by RT-qPCR and western blot assays (n=3 per group). B, YBX1 mRNA levels in stable silenced cells were measured by RT-qPCR (n=3 per group). C, YBX1 protein levels stable silenced cells were measured by western blot assays.

**Figure S3 Exogenous Flag-YBX1 expression in HK1 cell.**

Flag-YBX1 protein was detected by western blot assay in lentivirus infected HK1 cells.
